# MeV+R: using MeV as a graphical user interface for Bioconductor applications in microarray analysis

**DOI:** 10.1186/gb-2008-9-7-r118

**Published:** 2008-07-24

**Authors:** Vu T Chu, Raphael Gottardo, Adrian E Raftery, Roger E Bumgarner, Ka Yee Yeung

**Affiliations:** 1 Department of Microbiology, University of Washington, Seattle, WA 98195, USA; 2 Department of Statistics, University of British Columbia, Vancouver, BC, V6T 1Z2, Canada; 3 Department of Statistics, University of Washington, Seattle, WA 98195, USA

## Abstract

We present MeV+R, an integration of the JAVA MultiExperiment Viewer program with Bioconductor packages. This integration of MultiExperiment Viewer and R is easily extensible to other R packages and provides users with point and click access to traditionally command line driven tools written in R. We demonstrate the ability to use MultiExperiment Viewer as a graphical user interface for Bioconductor applications in microarray data analysis by incorporating three Bioconductor packages, RAMA, BRIDGE and iterativeBMA.

## Abstract 2

MeV+R provides users with point-and-click access to traditionally command-line-driven tools written in R.

## Rationale

While microarray technology has given biologists unprecedented access to gene expression data, reliable and effective data analysis remains a difficult problem. There are many freely or commercially available software packages, but biologists are often faced with trading off power and flexibility for usability and accessibility. In addition to the potentially prohibitive costs, researchers using commercial software tools may find themselves waiting for state-of-the-art algorithms to be implemented with the packages. The Bioconductor project [1,2] is an open source software project that provides a wide range of statistical tools primarily based on the R programming environment and language [3,4]. Taking advantage of R's powerful statistical and graphical capabilities, developers have created and contributed numerous Bioconductor packages to solve a variety of data analysis needs. The use of these packages, however, requires a basic understanding of the R programming/command language and an understanding of the documentation accompanying each package. The primary users of R and the Bioconductor packages have been computational scientists, statisticians and the more computationally oriented biologists. However, in our experience, many biologists find themselves uncomfortable issuing command lines in a terminal. Hence, there is a need for a graphical user interface (GUI) for Bioconductor packages that will allow biologists easy access to data analytical tools without learning the command line syntax. The tcltk package in R adds GUI elements to R by allowing programmers to write GUI-driven modules by embedding Tk commands into the R language [5]. There are also GUIs developed for basic statistical analysis in R, such as the R Commander [6] and windows-based SciViews [7]. However, these GUIs are not designed for microarray analysis. There are Bioconductor packages, such as limmaGUI [8], affylmGUI [9] and OLINgui [10] that are built on the R tcltk package to provide GUIs. LimmaGUI and affylmGUI provide GUIs for the analysis of designed experiments and the assessment of differential expression for two-color spotted microarrays and single-color Affymetrix data, respectively. OLINgui provides a GUI for the visualization, normalization and quality testing of two-channel microarray data. However, no such GUIs are available for the majority of Bioconductor packages. In addition, since each Bioconductor package is often written by a different research group, there is generally no uniformity in the look and feel of the GUIs available for the different packages. Hence, the end user may not be able to easily transfer experience gained with one analysis tool to the use of another.

An alternative microarray data analysis tool is the MultiExperiment Viewer (MeV), a component of the TM4 suite of microarray analysis tools [11]. MeV has a user-friendly GUI designed with the biological community in mind. MeV is an open source Java application with a simple to learn, easy to use GUI. It comes with many popular microarray analytical algorithms for clustering, visualization, classification and biological theme discovery, such as hierarchical clustering [12] and Expression Analysis Systematic Explorer (EASE) [13]. MeV was carefully designed to provide an application programming interface (API), thus allowing straightforward contributions by the community. MeV is hosted at SourceForge [14] in a concurrent versions system repository. As such, frequent builds of the source code are made possible, greatly reducing the lag time between version releases.

In this paper, we present MeV+R, which is an effort to provide more consistent and well-integrated GUIs for Bioconductor packages by using MeV as a 'wrapper' application for Bioconductor methods. Our work brings the best of both worlds together: providing state-of-the-art statistical algorithms from Bioconductor through the open source and easy to use MeV graphical interface to the biomedical community. MeV+R has many advantages, including platform independence, a well-defined modular API, and a point and click GUI that is easy to learn and use. We demonstrate the successful integration and advantages of three Bioconductor packages (RAMA [15], BRIDGE [16], and iterativeBMA [17]) over existing tools in the MeV environment through case studies. The underlying framework that we used to integrate these Bioconductor packages with MeV is easily extensible to other analysis tools developed in R. The software, documentation and a tutorial are publicly available from our project home page [18].

## Implementation

**Figure 1:**
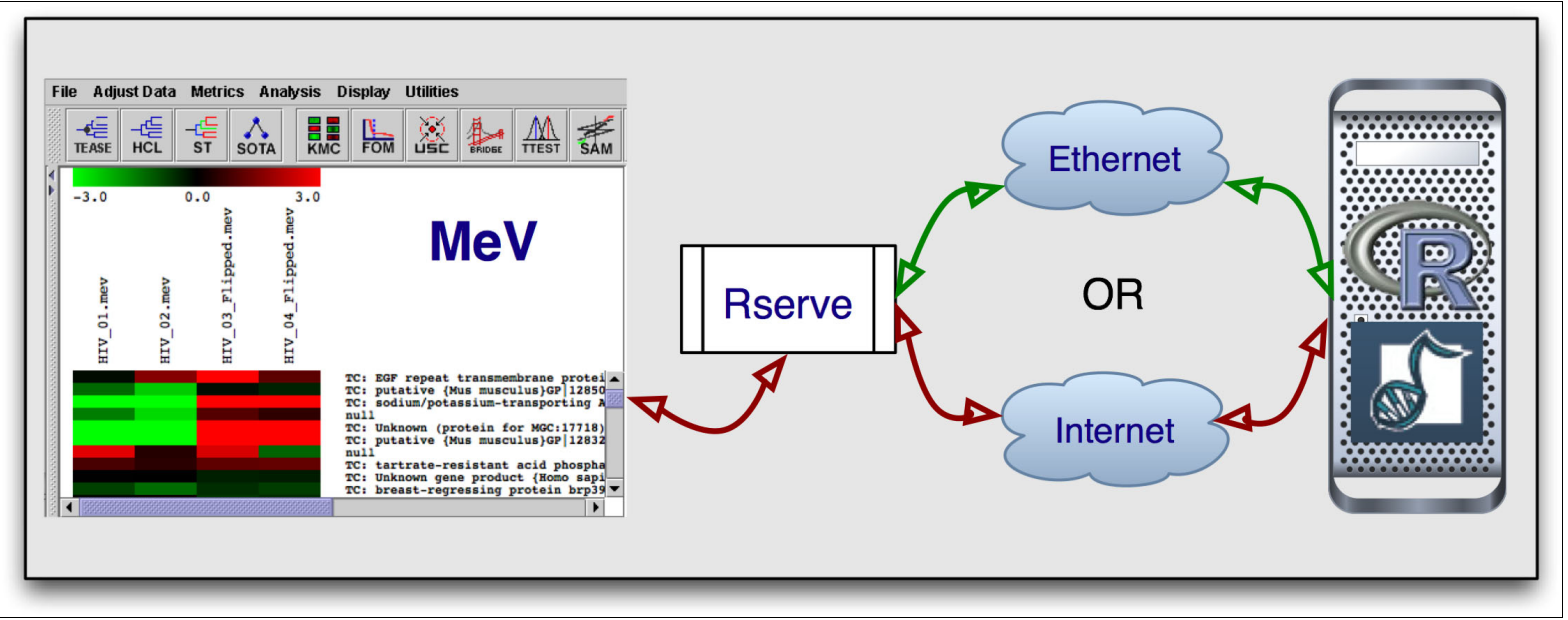
Our integration effort is composed of three separate entities: MeV as the GUI, Rserve as the communication layer, and R as the language and environment in which the analysis packages run.

Our integration effort is composed of three separate entities (Figure 1). MeV provides the graphical user interface while Rserve serves as the communication layer and R is the language and environment in which the analysis packages run. Rserve is a TCP/IP server that allows various languages to use the facilities of R without the need to initialize R or link against an R library [19]. In other words, we use R as the back end to run Bioconductor packages through the use of Rserve. Rserve is open source, freely available [20], and licensed under GPL.

As such, Java, Rserve, and R must all be installed on the user's computer, and we provide an automated installer on our project web site. Furthermore, Rserve needs to be running to be used. However, R does not need to be started. Since Rserve works through TCP/IP, it can run on the user's own machine, on an internal network or over the internet. By default, our code assumes Rserve to be running on the local host, but the user can change, add and save additional new hosts using a pull down menu. Once a connection is established, the Java code in MeV converts the user's data from the MeV data structure to the R format and loads it into R. The appropriate R libraries are loaded followed by the R commands that are necessary to initiate the analysis. Upon completion, the returned data from R are explicitly called back into MeV and presented to the user.

We have incorporated three Bioconductor packages, RAMA [15], BRIDGE [16], and iterativeBMA [17], into MeV to illustrate the successful MeV+R integration. The Robust Analysis of MicroArray (RAMA) algorithm computes robust estimates of expression intensities from two-color microarray data, which typically consist of a few replicates and potential outliers [15]. RAMA also takes advantage of dye swap experimental designs. Bayesian Robust Inference for Differential Gene Expression (BRIDGE) is a robust algorithm that selects differentially expressed genes under different experimental conditions on both one- and two-color microarray data [16]. Both RAMA and BRIDGE make use of a computationally intensive technique called Markov Chain Monte Carlo for parameter estimation, and it is non-trivial to re-implement these algorithms in Java. Hence, we took advantage of our previous development work by simply using MeV as an interface to the Bioconductor packages. The iterative BMA algorithm is a multivariate gene selection and classification algorithm, which considers multiple genes simultaneously and typically leads to a small number of relevant genes to classify microarray data [17]. The iterativeBMA Bioconductor package implements the iterative BMA algorithm as previously described [17] in R, and its implementation is part of our current integration effort. Both RAMA and BRIDGE are included in the latest release of MeV (version 4.1), and iterativeBMA will be included in future releases. The user interfaces, usage and case studies for RAMA, BRIDGE and iterativeBMA are briefly described below. Detailed documentation is included with the software distribution [21] as well as linked in the MeV application. Help pages are also available as Help Dialogs accessed via buttons on the MeV dialog boxes. Our MeV+R implementation is publicly available and runs on Windows, Mac OS X and Linux.

## Integrated Bioconductor packages: description and user interfaces

### RAMA: Robust Analysis of MicroArrays

RAMA uses a Bayesian hierarchical model for the robust estimation of cDNA microarray intensities with replicates. This is highly relevant for replicated microarray experiments because even one outlying replicate (such as due to scratches or dust) can have a disastrous effect on the estimated signal intensity. Outliers are modeled explicitly using a t-distribution, which is more robust than the usual Gaussian model. Our model borrows strength from all the genes to decide if a measurement is an outlier, and hence it is better at detecting outliers based on a small number of replicate measurements than other classical robust estimators. Our algorithm uses Markov Chain Monte Carlo for parameter estimation, and addresses classical issues such as design effects, normalization, transformation, and nonconstant variance. Please refer to [15] for a detailed description of the algorithm.

#### User interface

The user can start RAMA by clicking 'Adjust Data' - 'Replicate Analysis' - '*RAMA*' from the MeV main menu. The RAMA dialog box is then displayed asking the user to label the arrays that were loaded into MeV with their appropriate dye color. At this time, the user is asked to make sure that Rserve is running. On a Win32 system, double clicking Rserve.exe accomplishes this. On a UNIX or Linux or Mac OS X system, the user issues the command 'R CMD Rserve' at a prompt. By default, RAMA will look on the local machine for an Rserve server. However, since Rserve is a TCP/IP server, the Rserve server can be a remote machine. The user is allowed to adjust a few advanced parameters, though suggested values are given as defaults. If an Rserve connection is successfully made, the location of Rserve is written to the user's MeV configuration file and will be available in later sessions. After clicking 'OK', the input data are sent to R. An indeterminate progress bar is displayed while RAMA runs - unfortunately, the architecture of RServe and the R Server do not allow for an accurate indication of the time remaining in an ongoing analysis. Once completed, the user is given a dialog box to save the results. The returned results will then replace the loaded data in a new Multiple Array Viewer (MAV). The old MAV is deleted. The user can then choose to continue using MeV as if the data were loaded through the native loading modules.

### BRIDGE: Bayesian Robust Inference for Differential Gene Expression

BRIDGE fits a robust Bayesian hierarchical model to test for differentially expressed genes on microarray data. It can be used with both two-color microarrays and single-channel Affymetrix chips. BRIDGE builds on the previous work of Gottardo *et al*. [15] by allowing each gene to have a different variance and the detection of differentially expressed genes under multiple (up to three in our current implementation) experimental conditions. Robust inference is accomplished by modeling outliers using a t-distribution, and hence BRIDGE is powerful even with a small number of samples (either biological or technical replicates) under each experimental condition. Parameter estimation is carried out using a novel version of Markov Chain Monte Carlo. The current implementation of BRIDGE does not handle missing values. Please refer to [16] for a detailed description of the model.

#### User interface

BRIDGE starts when a user clicks the '*BRIDGE*' button in the toolbar located on top of the MeV window. The user is once again presented with a dialog box similar to that of RAMA asking for the dye labeling identity of each loaded slide. The user is offered the option to adjust the advanced parameters and to establish an Rserve connection. After clicking OK, the input data are sent to R. An indeterminate progress bar is displayed while BRIDGE runs. The results are presented to the user in three formats: heat maps, expression graphs or tables. In each format, the genes for which there is strong evidence of differential expression are identified as 'Significant Genes', defined by the posterior probability being above 0.5.

### IterativeBMA: Iterative Bayesian Model Averaging

The iterativeBMA algorithm is a multivariate technique for gene selection and classification of microarray data. Bayesian Model Averaging (BMA) takes model uncertainty into consideration by averaging over the predicted probabilities based on multiple models, weighted by their posterior model probabilities [22]. The most commonly used BMA algorithm is limited to data in which the number of variables is greater than the number of responses, and the algorithm is inefficient for datasets containing more than 30 genes (variables). In the case of classifying samples using microarray data, there are typically thousands or tens of thousands of genes (variables) under a few dozen samples (responses). In the iterative BMA algorithm, we start by ranking the genes using the ratio of between-group to within-group sum of squares (BSS/WSS) [23]. In this initial preprocessing step, genes with large BSS/WSS ratios (that is, genes with relatively large variation between classes and relatively small variation within classes) receive high rankings. We then apply the traditional BMA algorithm to the 30 top ranked genes, and remove genes with low posterior probabilities. Genes from the rank ordered BSS/WSS ratios are then added to the set of genes to replace genes with low probabilities. These steps of gene swaps and iterative applications of BMA are repeated until all genes are subsequently considered. We have previously shown that the iterative BMA algorithm selects small numbers of relevant genes, achieves high prediction accuracy, and produces posterior probabilities for the predictions, selected genes and models [17].

The iterativeBMA Bioconductor package implements the iterative BMA algorithm described in Yeung *et al*. [17] (previously implemented in Splus) when there are two classes. It is part of the original work for this publication. The user documentation (vignette) is included in the package.

#### User interface

We have integrated the iterativeBMA Bioconductor package in MeV. IterativeBMA starts after the user clicks on the 'iBMA' icon on top of the MeV window. The current implementation of the iterativeBMA Bioconductor package is limited to only two classes. After loading the data, the user is asked to label the two classes. The default labels for the two classes are 0 and 1, respectively. In the same dialog box, the user is asked to establish an Rserve connection. The user is also given the option of specifying advanced parameters for the analysis. The next dialog box asks the user to assign labels to each of the samples in the data, either by using a pull-down menu or loading an assignment file. At this point, if Rserve is not already running, the user is reminded to start the connection. Then, the data and the parameters are sent to R, and a progress bar is shown warning the user that the computation could take a long time. After the iterativeBMA Bioconductor package finishes running, the following analysis results are displayed: the predicted probability and class for each test sample; the posterior probabilities of the selected genes sorted in descending order; the posterior probabilities of the selected models sorted in descending order; and the heatmaps of the selected genes in both classes.

## Case studies illustrating the merits of the integrated Bioconductor packages

In this section, we compare the performance of the integrated Bioconductor packages (RAMA, BRIDGE and iterativeBMA) to existing tools in MeV in order to illustrate the merits of the integrated packages. In addition, we demonstrate that our MeV+R modules can be used together with other MeV modules in the integrated analysis of microarray data, hence, extending the capabilities of MeV.

### RAMA: Robust Analysis of MicroArrays

We compared the microarray gene intensities estimated using RAMA to that of the log ratios over intensities averaged over all the replicates on two microarray datasets and the results are summarized in Table 1. The first dataset is a subset of the HIV data [24] consisting of the expression levels of 1,028 transcripts, including 13 positive controls and 24 negative controls, in CD4-T-cell lines at time t = 1 hour after infection with HIV virus type 1 hybridized to two-color cDNA arrays. The experimental design consists of four technical replicates and balanced dye swap in which two of the four replicates were hybridized with Cy3 for the control and Cy5 for the treatment and then the dyes were reversed on the other two replicates. The second dataset is a subset of the like and like data [15] consisting of 1,000 genes over four experiments using the same RNA preparation isolated from a HeLa cell line on four different microarray slides. Since the same RNA was used in both channels, no genes from these data should show any differential expression. Both sample datasets are available on our project web site and are included as part of our MeV+R package release.

**Table 1:** Comparing the results of RAMA to the averaged log ratios on the HIV data and the like and like data

Data	Benchmark	RAMA	Averaged log ratio
HIV data	13 positive controls	All 13 positive controls have log ratios >1	All 13 positive controls have log ratios >1
	24 negative controls	All 24 negative controls have log ratios <1	3 negative controls have log ratios >1
Like and like data	No genes expected to be differentially expressed	All log ratios <1	6 genes with log ratios >1

RAMA produced the desired results on both datasets while the averaged log ratio produced three and six false positives, respectively, on these two datasets.

**Figure 2:**
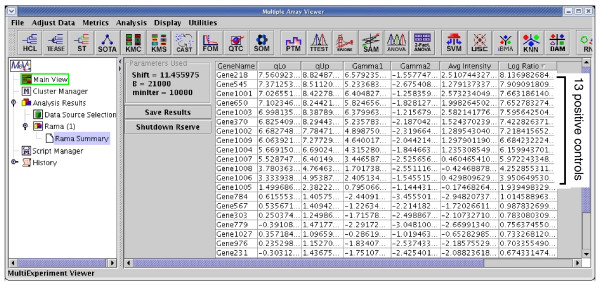
The results of applying RAMA to the HIV data. The log ratios computed from RAMA are sorted in descending order, and the top 13 genes with log ratios greater than one are the positive controls.

Figure 2 shows the log ratios of all genes sorted in descending order after applying RAMA integrated in MeV+R to the HIV data. As shown in Figure 2, the log ratios (to base 2) computed with the robust intensities estimated using RAMA for all 13 positive controls are all greater than one. The log ratios from RAMA for all 24 negative controls are smaller than one (data not shown in Figure 2). On the contrary, computing the log ratios by simply averaging the gene intensities over the four replicates produces log ratios greater than one for three negative controls. Applying RAMA to the like and like data produces no log ratio greater than one as desired since we do not expect any differentially expressed genes. On the contrary, the average log ratio of gene intensities yields six genes with log ratios greater than one. Please refer to the supplementary material [18] for the details of our case studies. To summarize, RAMA produced the desired results on both datasets while the averaged log ratio produced three and six false positives, respectively, on these two datasets.

### BRIDGE: Bayesian Robust Inference for Differential Gene Expression

**Figure 3:**
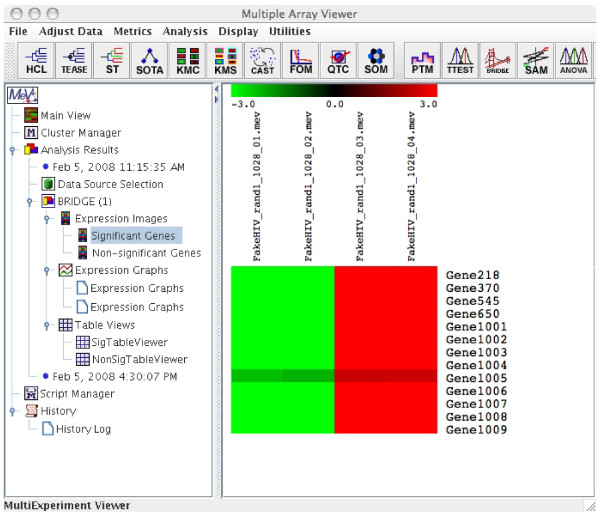
The significant genes identified by applying BRIDGE to the HIV data.

We compared the differentially expressed genes identified using BRIDGE, *t*-test and SAM (Significance Analysis of Microarrays) [25] as implemented in MeV on two datasets. Applying BRIDGE to the HIV data described in the previous section identified all 13 positive controls as 'significant' genes (Figure 3). On the other hand, applying the one-sample *t*-test as implemented in MeV to the same HIV data identified a total of 14 significant genes, including all 13 positive controls and one negative control using a *p*-value cut-off of 0.01 without any Bonferroni correction. Using a *p*-value cut-off of 0.05 and standard Bonferroni correction, the one-sample *t*-test identified only one significant gene (which is one of the 13 positive controls) and incorrectly assigned the remaining 12 positive controls as 'insignificant'. Similarly, using one-sample SAM as implemented in MeV identified 12 out of 13 positive controls using default parameters.

The second dataset we used comprises the Affymetrix U133 spike-in data [26], which consists of three technical replicates of 14 separate hybridizations of 42 spiked transcripts in a complex human background at varying concentrations. Thirty of the spikes are isolated from a human cell line, four spikes are bacterial controls, and eight spikes are artificially engineered sequences believed to be unique in the human genome. The data were preprocessed using GCRMA [27], resulting in a dataset of 22,300 genes across 42 samples. In addition to the original 42 spiked-in genes, we included an additional 20 genes that consistently showed significant differential expression across the array groups and an additional three genes containing probe sequences exactly matching those for the spiked-in genes [28,29]. As a result, our expanded spiked-in gene list contains 65 entries in total. We used a subset of this spiked-in data consisting of 1,059 genes that include all 65 spiked-in genes across two samples in triplicate. In our comparison, only the 65 spiked-in genes should be identified as differentially expressed.

BRIDGE identified 45 differentially expressed genes on this data subset. All of these 45 genes identified by BRIDGE are spiked-in genes. On the other hand, the *t*-test with a *p*-value cut-off of 0.01 without any correction for multiple comparison identified a total of 33 significant genes, of which 31 were spiked-in genes. Using a *p*-value cut-off of 0.05 and the standard Bonferroni correction, the *t*-test identified only four significant genes (which are among the spiked-in genes). SAM identified eight spiked-in genes as differentially expressed.

Our comparison results are summarized in Table 2. We have shown that BRIDGE is the only tool that successfully identified all 13 positive controls as 'significant' on the HIV data. In addition, BRIDGE identified the highest number of true positives (spiked-in genes) without any false positives on the Affymetrix spike-in data.

**Table 2:** Comparing the results of BRIDGE to *t*-test and SAM on the HIV data and the Affymetrix spike-in data

			*t*-test		
				
Dataset	Benchmark	BRIDGE	*p*-value cut-off 0.01, no correction	*p*-value cut-off 0.05, standard Bonferroni correction	SAM
HIV data	13 positive controls, 24 negative controls				
DE		13	14	1	12
TP		**13**	**13**	1	12
FP		**0**	1	**0**	**0**
Affymetrix spike-in data	65 spike-in genes				
DE		45	33	4	8
TP		**45**	31	4	8
FP		**0**	2	**0**	**0**

For each dataset and each method, the number of differentially expressed (DE) genes, true positives (TP) and false positives (FP) are shown. For each dataset, the maximum TP and the minimum FP across all methods are shown in bold. BRIDGE produced the best results on both datasets in identifying the highest number of true positives without any false positives.

### IterativeBMA: Iterative Bayesian Model Averaging

**Figure 4:**
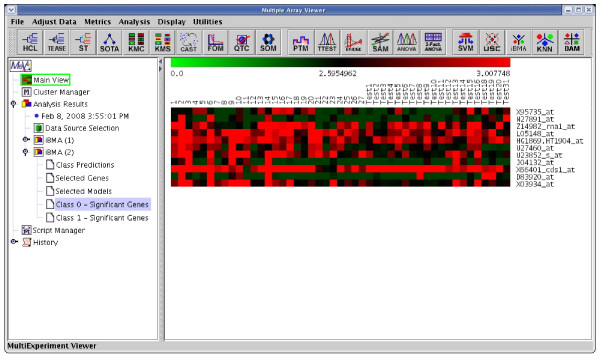
The results of applying iterativeBMA to the leukemia data. A heatmap showing the selected genes from iterativeBMA under the training samples labeled as class 0 and the test samples assigned to class 0 by the algorithm.

**Figure 5:**
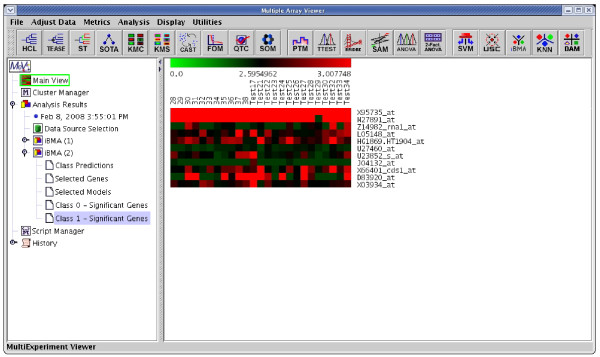
The results of applying iterativeBMA to the leukemia data. A heatmap showing the selected genes from iterativeBMA under the training samples labeled as class 1 and the test samples assigned to class 1 by the algorithm.

We compared the performance of iterativeBMA (abbreviated as iBMA in our MeV+R implementation) to KNN (k-nearest neighbor) [30] and USC (Uncorrelated Shrunken Centroid) [31] implemented in MeV using the well-studied leukemia data [32]. We used the filtered leukemia dataset, which consists of 3,051 genes, 38 samples in the training data and 34 samples in the test set. The data consist of samples from patients with either acute lymphoblastic leukemia (ALL) or acute myeloid leukemia (AML). On the leukemia data, iterativeBMA produced 2 classification errors using 11 selected genes over 11 models (Figures 4 and 5). On the other hand, KNN does not have a gene selection procedure and produced 2 classification errors using all 3,051 genes. Similarly, USC produced 2 classification errors using 51 selected genes.

The second dataset we used is the breast cancer prognosis dataset [33], which consists of 4,919 genes with 76 samples in the training set, and 19 samples in the test set [17]. The patient samples are divided into two categories: the good prognosis group (patients who remained disease free for at least five years) and the poor prognosis group (patients who developed distant metastases within five years). The iterativeBMA algorithm produced three classification errors using four genes averaged over three models. On the other hand, KNN does not have a gene selection procedure and produced five classification errors using all genes. Similarly, USC produced four classification errors using 662 genes.

Our results are summarized in Table 3. On the breast cancer prognosis data, iterativeBMA produced higher prediction accuracy using much fewer genes. On the leukaemia data, iterativeBMA produced comparable prediction accuracy using much fewer genes.

**Table 3:** Comparing the results of iterativeBMA to KNN and USC on the leukemia data and the breast cancer prognosis data

Data	Size of data	iterativeBMA	KNN	USC
Leukemia data [32]	38 training samples	**11 genes**	3,051 genes	51 genes
	34 test samples	2 errors	2 errors	2 errors
Breast cancer prognosis data [33]	76 training samples	**4 genes**	4,919 genes	662 genes
	19 test samples	**3 errors**	5 errors	4 errors

The number of selected genes and the number of classification errors are shown for each method. For each dataset, the smallest number of genes and the smallest number of classification errors across all three methods are shown in bold. On the leukemia data, iterativeBMA produced the same number of classification errors using much fewer genes. On the breast cancer prognosis data, iterativeBMA produced fewer errors using much fewer genes.

### Using other MeV modules in an integrated data analysis

The previous sub-sections showed that our MeV+R modules achieved superior performance when compared to other existing tools implemented in MeV. Here we demonstrate how the R packages that we incorporated into MeV can be used in combination with other existing tools in MeV. This illustrates the fact that the MeV+R framework has extended the capabilities of MeV, and that using these R packages through the MeV GUI adds value to the integrated analysis of microarray data.

**Figure 6:**
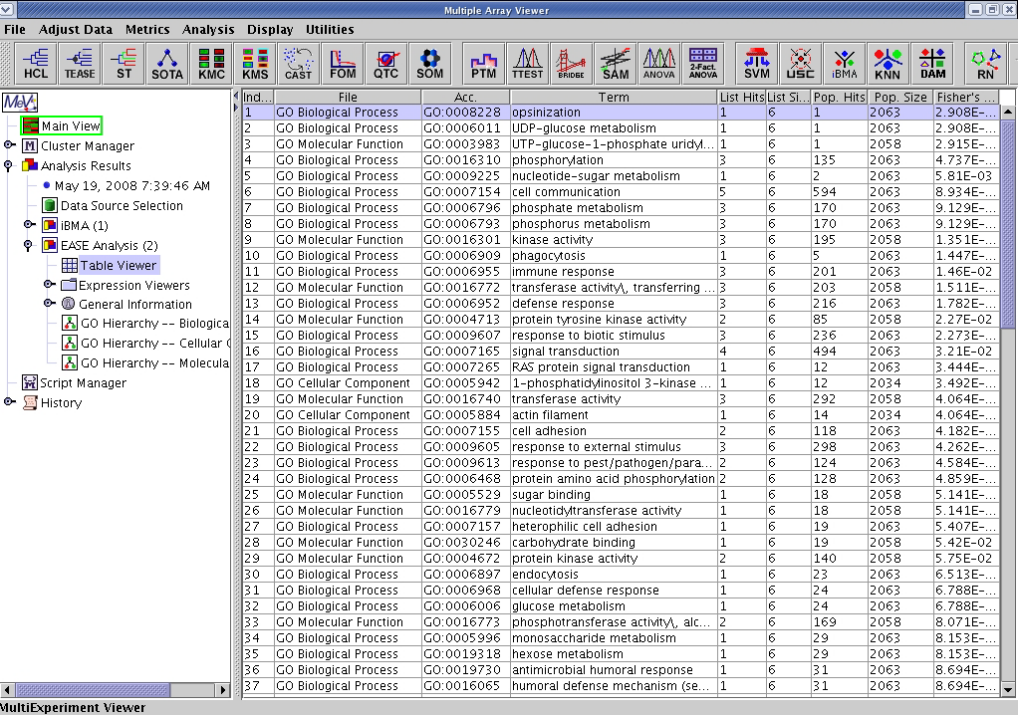
The results of applying EASE to the 11 genes selected by iterativeBMA on the leukemia data.

In this case study, we will follow-up on the results from applying the iterativeBMA algorithm to the leukemia data [32]. The iterativeBMA algorithm is a multivariate gene selection method designed to select a small set of predictive genes for the classification of microarray data. In the case of the leukemia data, the iterativeBMA algorithm selected 11 genes that produced two classification errors on the 34-sample test set. It would be interesting to identify the biological theme in this 11-gene list. Towards this end, we applied EASE [13] as implemented in MeV to determine the over-represented Gene Ontology categories in this gene list relative to all the genes on the microarray. Figure 6 shows the tabular view from the EASE analysis.

Since iterativeBMA identifies a small set of predictive genes for classification, other genes that exhibit similar expression patterns to the selected genes are likely of biological interest. For example, we would like to explore the gene with the highest posterior probability 'X95735_at' from the iterativeBMA analysis on the leukemia data [32]. We applied PTM (Template Matching) [34] as implemented in MeV to identify genes that are highly correlated with 'X95735_at'. Using a *p*-value threshold of 0.0001, PTM identified 209 genes that are highly correlated with 'X95735_at'. Our next task was to find the biological theme among these 209 genes, so we applied EASE and TEASE (Tree-EASE). TEASE is a combined analytical tool for hierarchical clustering and EASE. TEASE computes the dendrogram using the hierarchical clustering method and displays the significantly enriched Gene Ontology categories for each subtree in the dendrogram. Please refer to the supplementary materials [18] for the details of our case studies.

## Incorporating additional R packages

We have developed a framework with built-in functions for the integration of Bioconductor packages into MeV. Detailed documentation of these built-in functions is provided on our project web site for software developers. Using this framework, we have integrated three Bioconductor packages (RAMA, BRIDGE and iterativeBMA) into MeV as proof of concept. To integrate additional Bioconductor packages into MeV, a software developer can simply call our built-in functions except for complex and non-standard data views.

## Conclusion

MeV+R is a convenient platform to provide biologists with point and click GUI access to Bioconductor packages. We have demonstrated the successful integration of Bioconductor and MeV through three Bioconductor packages, RAMA, BRIDGE and iterativeBM, and that the incorporated Bioconductor packages produced superior results in the analysis of microarray data compared to existing tools in MeV. Additional Bioconductor packages are straightforward to add: the framework for moving data from MeV to R and back is generalized for code re-use, and each new package will merely require the development of a GUI for input and output.

## Abbreviations

API, application programming interface; BMA, Bayesian Model Averaging; BRIDGE, Bayesian Robust Inference for Differential Gene Expression; BSS/WSS, ratio of between-group to within-group sum of squares; EASE, Expression Analysis Systematic Explorer; GUI, graphical user interface; iterativeBMA, iterative Bayesian Model Averaging; KNN, k-nearest neighbor; MAV, Multiple Array Viewer; MeV, MultiExperiment Viewer; PTM, Template Matching; RAMA, Robust Analysis of MicroArray; SAM, Significance Analysis of Microarrays; USC, Uncorrelated Shrunken Centroid.

## Authors' contributions

VC carried out the software implementation, and drafted part of the initial manuscript. RG and AER designed and wrote the Bioconductor packages RAMA and BRIDGE, and assisted in incorporating these packages into MeV. REB conceived of the study, and designed and coordinated the project. KYY participated in the design and coordination of the study, wrote the iterativeBMA Bioconductor package, carried out the case studies and prepared the manuscript. All authors read and approved the final manuscript.
